# Supplementary material for: Status and trends of giant clam populations demonstrate the effectiveness of village-based protection in American Sāmoa
Source: PeerJ. 2025 Nov 14;13:e20290. doi: 10.7717/peerj.20290 (PMC12622233; doi:10.7717/peerj.20290)
Supplement: Supplemental Information 1 — Survey site names have been removed and GPS coordinates are limited to one decimal place to protect sensitive giant clam habitats from overharvesting. Additional supporting data are available upon reasonable request from the authors, but full access to site-specific information is restricted due to legal and conservation concerns. Table adapted from Green & Craig (1999) and Green et al. (2022). [file peerj-13-20290-s001.docx]

| **Island** | **Sector** | **Level of Protection** | **GPS Co-ordinates** | | **Number of Transects** | | | |
| --- | --- | --- | --- | --- | --- | --- | --- | --- |
|  |  |  | **Latitude** | **Longitude** | **1994/95** | **2002** | **2018** | **2022/24** |
| Tutuila | NE | Remote | -14.3 | -170.6 | 5 | 3 | 3 | 3 |
|  |  | Remote | -14.3 | -170.6 | 5 | 3 | 3 | 3 |
|  |  | Existing Government | -14.2 | -170.7 | 5 | 3 | 3 | 3 |
|  | NW | Remote | -14.3 | -170.8 | 5 | 3 | 3 | 3 |
|  |  | Village Protected | -14.3 | -170.8 | - | - | - | 3 |
|  |  | Village Protected | -14.3 | -170.8 | 5 | 3 | 3 | 3 |
|  |  | Village Protected | -14.3 | -170.7 | 5 | 3 | 3 | 9 |
|  | Pago Pago Harbor | Existing Government | -14.3 | -170.7 | 5 | 3 | 3 | 3 |
|  |  | Existing Government | -14.3 | -170.7 | 5 | 3 | 3 | 3 |
|  |  | Existing Government | -14.3 | -170.7 | 5 | 3 | 3 | 3 |
|  |  | Existing Government | -14.3 | -170.7 | 5 | 3 | 3 | 3 |
|  |  | Existing Government | -14.3 | -170.7 | 5 | 3 | 3 | 3 |
|  | SE | Existing Government | -14.3 | -170.6 | 5 |  | 3 | 3 |
|  |  | Existing Government | -14.3 | -170.6 | 5 | 3 | 3 | 3 |
|  |  | Existing Government | -14.3 | -170.7 | 5 | 3 | 3 | 3 |
|  |  | Existing Government | -14.3 | -170.7 | 5 | 3 | 3 | 3 |
|  | SW | Existing Government | -14.3 | -170.8 | 5 | 3 | 3 | 3 |
|  |  | Federal No Take | -14.4 | -170.8 | 5 | 3 | 3 | 3 |
|  |  | Existing Government | -14.3 | -170.8 | 5 | 3 | 3 | 3 |
|  | National Park | Subsistence & Remote | -14.3 | -170.7 | - | - | - | 3 |
|  |  | Subsistence & Remote | -14.3 | -170.7 | - | - | - | 3 |
|  |  | Subsistence & Remote | -14.3 | -170.7 | - | - | - | 3 |
|  |  | Subsistence & Remote | -14.3 | -170.7 | - | - | - | 3 |
|  |  | Subsistence & Remote | -14.2 | -170.7 | - | - | - | 3 |
|  |  | Subsistence & Remote | -14.2 | -170.7 | - | - | - | 3 |
|  |  | Subsistence & Remote | -14.3 | -170.7 | - | - | - | 3 |
|  |  | Subsistence & Remote | -14.3 | -170.7 | - | - | - | 3 |
|  |  | Subsistence & Remote | -14.2 | -170.7 | - | - | - | 3 |
| Aunu‘u | S | Federal No Take | -14.3 | -170.6 | 5 | 3 | 3 | 3 |
| Ofu-Olosega | N | Existing Government | -14.2 | -169.6 | 5 | 5 | 3 | 3 |
|  |  | Existing Government | -14.2 | -169.7 | - | - | - | 3 |
|  |  | Existing Government | -14.2 | -169.7 | - | - | - | 3 |
|  |  | Existing Government | -14.2 | -169.6 | 5 | 5 | 3 | 3 |
|  | S | Subsistence | -14.2 | -169.7 | - | 5 | - | 3 |
|  |  | Existing Government | -14.2 | -169.7 | 5 | 5 | 3 | 3 |
|  |  | Existing Government | -14.2 | -169.6 | 5 | 5 | 3 | 3 |
| Ta‘ū | N | Existing Government | -14.2 | -169.4 | 5 | 5 | - | 3 |
|  |  | Existing Government | -14.2 | -169.5 | - | - | - | 3 |
|  |  | Remote | -14.2 | -169.5 | - | - | - | 3 |
|  |  | Remote | -14.2 | -169.5 | 5 | 5 | - | 3 |
|  | S | Remote | -14.3 | -169.5 | 5 | 5 | 3 | 3 |
|  |  | Subsistence | -14.3 | -169.4 | - | - | - | 3 |
|  |  | Remote | -14.2 | -169.5 | 5 | 5 | 3 | 3 |
| *Muliāva*  (Rose Atoll)  Bombies | NE | Inaccessible | -14.5 | -168.1 |  |  |  | X |
|  |  | Inaccessible | -14.5 | -168.1 |  |  |  | X |
|  |  | Inaccessible | -14.5 | -168.1 |  |  |  | X |
|  |  | Inaccessible | -14.5 | -168.1 |  |  |  | X |
|  | SE | Inaccessible | -14.5 | -168.1 |  |  |  | X |
|  |  | Inaccessible | -14.5 | -168.1 |  |  |  | X |
|  |  | Inaccessible | -14.5 | -168.1 |  |  |  | X |
|  |  | Inaccessible | -14.5 | -168.1 |  |  |  | X |
|  |  | Inaccessible | -14.5 | -168.1 |  |  |  | X |
|  |  | Inaccessible | -14.5 | -168.1 |  |  |  | X |
|  |  | Inaccessible | -14.5 | -168.1 |  |  |  | X |
|  |  | Inaccessible | -14.5 | -168.1 |  |  |  | X |
|  |  | Inaccessible | -14.5 | -168.1 |  |  |  | X |
|  |  | Inaccessible | -14.5 | -168.1 |  |  |  | X |
|  |  | Inaccessible | -14.5 | -168.1 |  |  |  | X |
|  |  | Inaccessible | -14.5 | -168.1 |  |  |  | X |
|  | SW | Inaccessible | -14.6 | -168.2 |  |  |  | X |
|  |  | Inaccessible | -14.6 | -168.2 |  |  |  | X |
|  |  | Inaccessible | -14.6 | -168.2 |  |  |  | X |
|  |  | Inaccessible | -14.6 | -168.2 |  |  |  | X |
|  |  | Inaccessible | -14.6 | -168.2 |  |  |  | X |
| *Muliāva*  (Rose Atoll)  Pinnacles | NW | Inaccessible | -14.5 | -168.2 |  |  |  | X |
|  |  | Inaccessible | -14.5 | -168.2 |  |  |  | X |
|  |  | Inaccessible | -14.5 | -168.2 |  |  |  | X |
|  | NE | Inaccessible | -14.5 | -168.2 |  |  |  | X |
|  |  | Inaccessible | -14.5 | -168.2 |  |  |  | X |
|  |  | Inaccessible | -14.5 | -168.2 |  |  |  | X |
|  | SW | Inaccessible | -14.6 | -168.2 |  |  |  | X |
|  |  | Inaccessible | -14.6 | -168.2 |  |  |  | X |
|  |  | Inaccessible | -14.6 | -168.2 |  |  |  | X |
