# Supplementary material for: Status and trends of giant clam populations demonstrate the effectiveness of village-based protection in American Sāmoa
Source: PeerJ. 2025 Nov 14;13:e20290. doi: 10.7717/peerj.20290 (PMC12622233; doi:10.7717/peerj.20290)
Supplement: Supplemental Information 2 [file peerj-13-20290-s002.docx]

| **Habitat Type** | **Island** | **1994/95** | **2002** | **2018** | **2022-2024** |
| --- | --- | --- | --- | --- | --- |
| **Reef Slope** | Tutuila | 13.33 | 44.44 | 40.74 | 95.24 |
|  | Aunu'u | 80.00 | 60.00 | 66.67 | 300.00 |
|  | Ofu | 260.00 | 233.33 | 50.00 | 133.33 |
|  | Olosega | 230.00 | 420.00 | 50.00 | 116.67 |
|  | Ta‘ū | 460.00 | 1535.00 | 350.00 | 730.48 |
| **Lagoon** | Ofu | 126.67 | 40.00 | - | - |
|  | Tutuila | - | - | - | 9.20 |
|  | Muliava | - | - | - | 25.00 |
