# Supplementary material for: Status and trends of giant clam populations demonstrate the effectiveness of village-based protection in American Sāmoa
Source: PeerJ. 2025 Nov 14;13:e20290. doi: 10.7717/peerj.20290 (PMC12622233; doi:10.7717/peerj.20290)
Supplement: Supplemental Information 3 — B) Mean density (clams/ha) of live giant clams surveyed on reef slopes (10 m) on Tutuila, American Sāmoa (2022-2024) delineated by type of protection. [file peerj-13-20290-s003.docx]

| **Archipelago-Wide** | | | | |
| --- | --- | --- | --- | --- |
| **Protection Type** | **Transect Count** | **Mean Clams / Ha** | **StdDev Clams / Ha** | **Std Error** |
| Existing Government | 70 | 101.05 | 194.20 | 23.21 |
| Federal No Take | 8 | 88.33 | 94.13 | 33.28 |
| Remote | 24 | 473.61 | 839.16 | 171.29 |
| Subsistence | 3 | 113.33 | 132.83 | 76.69 |
| Subsistence & Remote | 9 | 200.00 | 138.44 | 46.15 |
| Village Protected | 9 | 119.26 | 107.52 | 35.84 |

| **Tutuila** | | | | |
| --- | --- | --- | --- | --- |
| **Protection Type** | **Transect Count** | **Mean Clams / Ha** | **StdDev Clams / Ha** | **Std Error** |
| Existing Government | 48 | 17.50 | 43.35 | 6.26 |
| Federal No Take | 4 | 50.00 | 57.74 | 28.87 |
| Remote | 12 | 43.89 | 57.73 | 16.67 |
| Subsistence & Remote | 9 | 200.00 | 138.44 | 46.15 |
| Village Protected | 9 | 119.26 | 107.52 | 35.84 |
