# Supplementary material for: Status and trends of giant clam populations demonstrate the effectiveness of village-based protection in American Sāmoa
Source: PeerJ. 2025 Nov 14;13:e20290. doi: 10.7717/peerj.20290 (PMC12622233; doi:10.7717/peerj.20290)
Supplement: Supplemental Information 4 — Various statistical results from our R code, using datasets variety of datasets (All years, All years except 2018, and 2022-2024). All code was run using RStudio (v2023.03.0+386). [file peerj-13-20290-s004.docx]

|  |  |  |  |  |  |  |
| --- | --- | --- | --- | --- | --- | --- |
| **2022-2024, Tutuila Only** | | | | | | |
| **Quadrant** | | | | | | |
| **anova_tut_quadrant <- aov(Clams...Hectare ~ Quadrant, data = tutuila_data)** | | | | | | |
|  | Df | Sum Sq | Mean Sq | F Value | Pr(>F) |  |
| Quadrant | 4 | 191160 | 47790 | 5.735 | 0.00128 | ** |
| Residuals | 33 | 274997 | 8333 |  |  |  |
| Significance codes | 0 ‘***’ | 0.001 ‘**’ | 0.01 ‘*’ | 0.05 ‘.’ | 0.1 ‘’ | 1 |
| Parameter |  | Eta2 |  | 95% | CI |  |
| Quadrant |  | 0.41 |  | [0.15, | 1.00] |  |
|  |  |  |  |  |  |  |
|  |  |  |  |  |  |  |
| **Protection** | | | | | | |
| **anova_tut_protection <- aov(Clams...Hectare ~ Protection, data = tutuila_data)** | | | | | | |
|  | Df | Sum Sq | Mean Sq | F Value | Pr(>F) |  |
| Protection | 4 | 258806 | 64702 | 10.3 | 1.59E-05 | *** |
| Residuals | 33 | 207352 | 6283 |  |  |  |
| Significance codes | 0 ‘***’ | 0.001 ‘**’ | 0.01 ‘*’ | 0.05 ‘.’ | 0.1 ‘’ | 1 |
| eta_squared(anova_tut_protection) | | |  |  |  |  |
| Parameter |  | Eta2 |  | 95% | CI |  |
| Protection2 |  | 0.56 |  | [0.32, | 1.00] |  |
|  |  |  |  |  |  |  |
|  |  |  |  |  |  |  |
| **Jurisdiction** | | | | | | |
| **anova_tut_Juris <- aov(Clams...Hectare ~ Jurisdiction, data = tutuila_data)** | | | | | | |
|  | Df | Sum Sq | Mean Sq | F Value | Pr(>F) |  |
| Jurisdiction | 2 | 156254.00000 | 78127.00000 | 8.82400 | 0.00079 | *** |
| Residuals | 35 | 309903.00000 | 8854.00000 |  |  |  |
| Significance codes | 0 ‘***’ | 0.001 ‘**’ | 0.01 ‘*’ | 0.05 ‘.’ | 0.1 ‘’ | 1 |
| Parameter | \| | Eta2 | \| | 95% | CI |  |
| Jurisdiction | \| | 0.34000 | \| | [0.12, | 1.00] |  |
|  |  |  |  |  |  |  |
|  |  |  |  |  |  |  |
| **Quadrant * Protection** | | | | | | |
| **anova_tut_quad_pro <- aov(Clams...Hectare ~ Quadrant * Protection2, data = tutuila_data)** | | | | | | |
|  | Df | Sum Sq | Mean Sq | F Value | Pr(>F) |  |
| Quadrant | 4 | 191160 | 47790 | 7.316 | 0.000436 | *** |
| Protection2 | 4 | 91810 | 22953 | 3.514 | 0.020195 | * |
| Quadrant:Protection2 | 3 | 13356 | 4452 | 0.682 | 0.571294 |  |
| Residuals | 26 | 169831 | 6532 |  |  |  |
| Significance codes | 0 ‘***’ | 0.001 ‘**’ | 0.01 ‘*’ | 0.05 ‘.’ | 0.1 ‘’ | 1 |
| Parameter | Gen | Eta2 |  | 95% | CI |  |
| Quadrant |  | 0.41 |  | [0.10, | 1.00] |  |
| Protection2 |  | 0.2 |  | [0.00, | 1.00] |  |
| Quadrant:Protection2 |  | 0.03 |  | [0.00, | 1.00] |  |
| Parameter | partial | Eta2 |  | 95% | CI |  |
| Quadrant |  | 0.53 |  | [0.25, | 1.00] |  |
| Protection2 |  | 0.35 |  | [0.04, | 1.00] |  |
| Quadrant:Protection2 |  | 0.07 |  | [0.00, | 1.00] |  |
|  |  |  |  |  |  |  |
| **Jurisdiction * Protection** | | | | | | |
| **anova_tut_juris_pro <- aov(Clams...Hectare ~ Jurisdiction * Protection2, data = tutuila_data)** | | | | | | |
|  | Df | Sum Sq | Mean Sq | F Value | Pr(>F) |  |
| Jurisdiction | 2 | 156254 | 78127 | 12.634 | 9.03E-05 | *** |
| Protection2 | 3 | 112011 | 37337 | 6.038 | 0.00223 | ** |
| Residuals | 32 | 197892 | 6184 |  |  |  |
| Significance codes | 0 ‘***’ | 0.001 ‘**’ | 0.01 ‘*’ | 0.05 ‘.’ | 0.1 ‘’ | 1 |
| Parameter | Gen | Eta2 |  | 95% | CI |  |
| Jurisdiction | \| | 0.34 | \| | [0.11, | 1.00] |  |
| Protection2 | \| | 0.24 | \| | [0.02, | 1.00] |  |
| Parameter | partial | Eta2 |  | 95% | CI |  |
| Jurisdiction | \| | 0.44 | \| | [0.21, | 1.00] |  |
| Protection2 | \| | 0.36 | \| | [0.11, | 1.00] |  |
|  |  |  |  |  |  |  |
|  |  |  |  |  |  |  |
| **Island * Year * Protection** | | | | | | |
| **aov_full <- aov(Dens ~ Island * Year * Protection, data = ReefSlope_allyears)** | | | | | | |
|  | Df | Sum Sq | Mean Sq | F Value | Pr(>F) |  |
| Quadrant | 4 | 191160 | 47790 | 7.316 | 4.36E-04 | *** |
| Jurisdiction | 2 | 30018 | 15009 | 2.298 | 0.120533 |  |
| Protection2 | 3 | 62625 | 20875 | 3.196 | 0.03999 | * |
| Quadrant:Jurisdiction | 1 | 4610 | 4610 | 0.706 | 0.408498 |  |
| Quadrant:Protection2 | 1 | 7914 | 7914 | 1.212 | 0.281118 |  |
| Residuals | 26 | 169831 | 6532 |  |  |  |
| Significance codes | 0 ‘***’ | 0.001 ‘**’ | 0.01 ‘*’ | 0.05 ‘.’ | 0.1 ‘’ | 1 |
| Parameter | Gen | Eta2 |  | 95% | CI |  |
| Quadrant |  | 0.41 |  | [0.10, | 1.00] |  |
| Jurisdiction |  | 0.06 |  | [0.00, | 1.00] |  |
| Protection2 |  | 0.13 |  | [0.00, | 1.00] |  |
| Quadrant:Jurisdiction |  | 9.89E-03 |  | [0.00, | 1.00] |  |
| Quadrant:Protection2 |  | 0.02 |  | [0.00, | 1.00] |  |
| Parameter | Gen | Eta2 |  | 95% | CI |  |
| Quadrant |  | 0.53 |  | [0.25, | 1.00] |  |
| Jurisdiction |  | 0.15 |  | [0.00, | 1.00] |  |
| Protection2 |  | 0.27 |  | [0.01, | 1.00] |  |
| Quadrant:Jurisdiction |  | 3.00E-02 |  | [0.00, | 1.00] |  |
| Quadrant:Protection2 |  | 0.04 |  | [0.00, | 1.00] |  |
|  |  |  |  |  |  |  |
|  |  |  |  |  |  |  |
| **All Years** | | | | | | |
| **Year** | | | | | | |
| **aov_year <- aov(Dens ~ Year, data = ReefSlope_allyears)** | | | | | | |
|  | Df | Sum Sq | Mean Sq | F Value | Pr(>F) |  |
| Year | 3 | 896035 | 298678 | 1.703 | 0.17 | . |
| Residuals | 119 | 20868060 | 175362 |  |  |  |
| Significance codes | 0 ‘***’ | 0.001 ‘**’ | 0.01 ‘*’ | 0.05 ‘.’ | 0.1 ‘’ | 1 |
| Parameter |  | Eta2 |  | 95% | CI |  |
| Year |  | 0.04 |  | [0.00, | 1.00] |  |
|  |  |  |  |  |  |  |
|  |  |  |  |  |  |  |
| **Island** | | | | | | |
| **aov_island <- aov(Dens ~ Island, data = ReefSlope_allyears)** | | | | | | |
|  | Df | Sum Sq | Mean Sq | F Value | Pr(>F) |  |
| Island | 4 | 8093702 | 2023425 | 17.47 | 2.79E-11 | *** |
| Residuals | 118 | 13670393 | 115851 |  |  |  |
| Significance codes | 0 ‘***’ | 0.001 ‘**’ | 0.01 ‘*’ | 0.05 ‘.’ | 0.1 ‘’ | 1 |
| Parameter |  | Eta2 |  | 95% | CI |  |
| Island |  | 0.37 |  | [0.25, | 1.00] |  |
|  |  |  |  |  |  |  |
|  |  |  |  |  |  |  |
|  |  |  |  |  |  |  |
| **Protection** | | | | | | |
| **aov_protection <- aov(Dens ~ Protection, data = ReefSlope_allyears)** | | | | | | |
|  | Df | Sum Sq | Mean Sq | F Value | Pr(>F) |  |
| Protection | 5 | 2622290 | 524458 | 3.206 | 0.0095 | ** |
| Residuals | 117 | 19141805 | 163605 |  |  |  |
| Significance codes | 0 ‘***’ | 0.001 ‘**’ | 0.01 ‘*’ | 0.05 ‘.’ | 0.1 ‘’ | 1 |
| Parameter |  | Eta2 |  | 95% | CI |  |
| Protection |  | 0.12 |  | [0.02, | 1.00] |  |
|  |  |  |  |  |  |  |
|  |  |  |  |  |  |  |
| **Island * Protection** | | | | | | |
| **aov_island_prot <- aov(Dens ~ Island * Protection, data = ReefSlope_allyears)** | | | | | | |
|  | Df | Sum Sq | Mean Sq | F Value | Pr(>F) |  |
| Island | 4 | 8093702 | 2023425 | 17.467 | 3.83E-11 | *** |
| Protection | 5 | 636048 | 127210 | 1.098 | 0.366 |  |
| Island:Protection | 2 | 175488 | 87744 | 0.757 | 0.471 |  |
| Residuals | 111 | 12858857 | 115846 |  |  |  |
| Significance codes | 0 ‘***’ | 0.001 ‘**’ | 0.01 ‘*’ | 0.05 ‘.’ | 0.1 ‘’ | 1 |
| Parameter | Gen | Eta2 |  | 95% | CI |  |
| Island |  | 0.37 |  | [0.24, | 1.00] |  |
| Protection |  | 0.03 |  | [0.00, | 1.00] |  |
| Island:Protection |  | 8.06E-03 |  | [0.00, | 1.00] |  |
| Parameter | partial | Eta2 |  | 95% | CI |  |
| Island |  | 0.39 |  | [0.26, | 1.00] |  |
| Protection |  | 0.05 |  | [0.00, | 1.00] |  |
| Island:Protection |  | 0.01 |  | [0.00, | 1.00] |  |
|  |  |  |  |  |  |  |
| **Year * Protection** | | | | | | |
| **aov_prot_year <- aov(Dens ~ Protection * Year, data = ReefSlope_allyears)** | | | | | | |
|  | Df | Sum Sq | Mean Sq | F Value | Pr(>F) |  |
| Protection | 5 | 2622290 | 524458 | 3.237 | 0.00932 | ** |
| Year | 3 | 888864 | 296288 | 1.829 | 0.14646 |  |
| Protection:Year | 10 | 1405179 | 140518 | 0.867 | 0.56594 |  |
| Residuals | 104 | 16847762 | 161998 |  |  |  |
| Significance codes | 0 ‘***’ | 0.001 ‘**’ | 0.01 ‘*’ | 0.05 ‘.’ | 0.1 ‘’ | 1 |
| Parameter | Gen | Eta2 |  | 95% | CI |  |
| Protection |  | 0.12 |  | [0.01, | 1.00] |  |
| Year |  | 0.04 |  | [0.00, | 1.00] |  |
| Protection:Year |  | 0.06 |  | [0.00, | 1.00] |  |
| Parameter | partial | Eta2 |  | 95% | CI |  |
| Protection |  | 0.13 |  | [0.02, | 1.00] |  |
| Year |  | 0.05 |  | [0.00, | 1.00] |  |
| Protection:Year |  | 0.08 |  | [0.00, | 1.00] |  |
|  |  |  |  |  |  |  |
| **Year * Island** | | | | | | |
| **aov_island_year <- aov(Dens ~ Island * Year, data = ReefSlope_allyears)** | | | | | | |
|  | Df | Sum Sq | Mean Sq | F Value | Pr(>F) |  |
| Island | 4 | 8093702 | 2023425 | 20.299 | 2.37E-12 | *** |
| Year | 3 | 657387 | 219129 | 2.198 | 0.0927 | . |
| Island:Year | 12 | 2745875 | 228823 | 2.296 | 0.0124 | * |
| Residuals | 103 | 10267131 | 99681 |  |  |  |
| Significance codes | 0 ‘***’ | 0.001 ‘**’ | 0.01 ‘*’ | 0.05 ‘.’ | 0.1 ‘’ | 1 |
| Parameter | Gen | Eta2 |  | 95% | CI |  |
| Island |  | 0.37 |  | [0.24, | 1.00] |  |
| Year |  | 0.03 |  | [0.00, | 1.00] |  |
| Island:Year |  | 0.13 |  | [0.00, | 1.00] |  |
| Parameter | partial | Eta2 |  | 95% | CI |  |
| v |  | 0.44 |  | [0.31, | 1.00] |  |
| Year |  | 0.06 |  | [0.00, | 1.00] |  |
| Island:Year |  | 0.21 |  | [0.03, | 1.00] |  |
|  |  |  |  |  |  |  |
| **Island * Year * Protection** | | | | | | |
| **aov_full <- aov(Dens ~ Island * Year * Protection, data = ReefSlope_allyears)** | | | | | | |
|  | Df | Sum Sq | Mean Sq | F Value | Pr(>F) |  |
| Island | 4 | 8093702 | 2023425 | 18.819 | 4.45E-11 | *** |
| Year | 3 | 657387 | 219129 | 2.038 | 0.1147 |  |
| Protection | 5 | 692744 | 138549 | 1.289 | 0.2766 |  |
| Island:Year | 12 | 2651132 | 220928 | 2.055 | 0.0289 | * |
| Island:Protection | 2 | 237496 | 118748 | 1.104 | 0.3362 |  |
| Year:Protection | 10 | 171462 | 17146 | 0.159 | 0.9984 |  |
| Island:Year:Protection | 2 | 228413 | 114206 | 1.062 | 0.3503 |  |
| Residuals | 84 | 9031759 | 107521 |  |  |  |
| Significance codes | 0 ‘***’ | 0.001 ‘**’ | 0.01 ‘*’ | 0.05 ‘.’ | 0.1 ‘’ | 1 |
| Parameter | Gen | Eta2 |  | 95% | CI |  |
| Island |  | 0.37 |  | [0.22, | 1.00] |  |
| Year |  | 0.03 |  | [0.00, | 1.00] |  |
| Protection |  | 0.03 |  | [0.00, | 1.00] |  |
| Island:Year |  | 0.12 |  | [0.00, | 1.00] |  |
| Island:Protection |  | 0.01 |  | [0.00, | 1.00] |  |
| Year:Protection |  | 7.88E-03 |  | [0.00, | 1.00] |  |
| Island:Year:Protection |  | 0.01 |  | [0.00, | 1.00] |  |
| Parameter | partial | Eta2 |  | 95% | CI |  |
| Island |  | 0.47 |  | [0.33, | 1.00] |  |
| Year |  | 0.07 |  | [0.00, | 1.00] |  |
| Protection |  | 0.07 |  | [0.00, | 1.00] |  |
| Island:Year |  | 0.23 |  | [0.01, | 1.00] |  |
| Island:Protection |  | 0.03 |  | [0.00, | 1.00] |  |
| Year:Protection |  | 0.02 |  | [0.00, | 1.00] |  |
| Island:Year:Protection |  | 0.02 |  | [0.00, | 1.00] |  |
|  |  |  |  |  |  |  |
|  |  |  |  |  |  |  |
|  |  |  |  |  |  |  |
| **All Years except 2018** | | | | | | |
| **Year** | | | | | | |
| **aov_year <- aov(Dens ~ Year, data = ReefSlope_allyears)** | | | | | | |
|  | Df | Sum Sq | Mean Sq | F Value | Pr(>F) |  |
| Year | 2 | 489763 | 244882 | 1.132 | 0.327 | . |
| Residuals | 95 | 20551438 | 216331 |  |  |  |
| Significance codes | 0 ‘***’ | 0.001 ‘**’ | 0.01 ‘*’ | 0.05 ‘.’ | 0.1 ‘’ | 1 |
| Parameter |  | Eta2 |  | 95% | CI |  |
| Year |  | 0.02 |  | [0.00, | 1.00] |  |
|  |  |  |  |  |  |  |
|  |  |  |  |  |  |  |
| **Island** | | | | | | |
| **aov_island <- aov(Dens ~ Island, data = ReefSlope_allyears)** | | | | | | |
|  | Df | Sum Sq | Mean Sq | F Value | Pr(>F) |  |
| Island | 4 | 8100574 | 2025143 | 14.55 | 2.88E-09 | *** |
| Residuals | 93 | 12940627 | 139147 |  |  |  |
| Significance codes | 0 ‘***’ | 0.001 ‘**’ | 0.01 ‘*’ | 0.05 ‘.’ | 0.1 ‘’ | 1 |
| Parameter |  | Eta2 |  | 95% | CI |  |
| Island |  | 0.38 |  | [0.24, | 1.00] |  |
|  |  |  |  |  |  |  |
|  |  |  |  |  |  |  |
| **Protection** | | | | | | |
| **aov_protection <- aov(Dens ~ Protection, data = ReefSlope_allyears)** | | | | | | |
|  | Df | Sum Sq | Mean Sq | F Value | Pr(>F) |  |
| Protection | 5 | 2903072 | 580614 | 2.945 | 0.0164 | * |
| Residuals | 92 | 18138129 | 197154 |  |  |  |
| Significance codes | 0 ‘***’ | 0.001 ‘**’ | 0.01 ‘*’ | 0.05 ‘.’ | 0.1 ‘’ | 1 |
| Parameter |  | Eta2 |  | 95% | CI |  |
| Protection |  | 0.14 |  | [0.02, | 1.00] |  |
|  |  |  |  |  |  |  |
|  |  |  |  |  |  |  |
| **Island * Protection** | | | | | | |
| **aov_island_prot <- aov(Dens ~ Island * Protection, data = ReefSlope_allyears)** | | | | | | |
|  | Df | Sum Sq | Mean Sq | F Value | Pr(>F) |  |
| Island | 4 | 8100574 | 2025143 | 14.63 | 3.73E-09 | *** |
| Protection | 5 | 759566 | 151913 | 1.097 | 0.368 |  |
| Island:Protection | 2 | 276313 | 138156 | 0.998 | 0.373 |  |
| Residuals | 86 | 11904748 | 138427 |  |  |  |
| Significance codes | 0 ‘***’ | 0.001 ‘**’ | 0.01 ‘*’ | 0.05 ‘.’ | 0.1 ‘’ | 1 |
| Parameter | Gen | Eta2 |  | 95% | CI |  |
| Island |  | 0.38 |  | [0.24, | 1.00] |  |
| Protection |  | 0.04 |  | [0.00, | 1.00] |  |
| Island:Protection |  | 0.01 |  | [0.00, | 1.00] |  |
| Parameter | partial | Eta2 |  | 95% | CI |  |
| Island |  | 0.4 |  | [0.26, | 1.00] |  |
| Protection |  | 0.06 |  | [0.00, | 1.00] |  |
| Island:Protection |  | 0.02 |  | [0.00, | 1.00] |  |
|  |  |  |  |  |  |  |
|  |  |  |  |  |  |  |
| **Year * Protection** | | | | | | |
| **aov_prot_year <- aov(Dens ~ Protection * Year, data = ReefSlope_allyears)** | | | | | | |
|  | Df | Sum Sq | Mean Sq | F Value | Pr(>F) |  |
| Protection | 5 | 2903072 | 580614 | 2.897 | 0.0185 | * |
| Year | 2 | 512495 | 256248 | 1.279 | 0.2839 |  |
| Protection:Year | 7 | 990247 | 141464 | 0.706 | 0.6671 |  |
| Residuals | 83 | 16635387 | 200426 |  |  |  |
| Significance codes | 0 ‘***’ | 0.001 ‘**’ | 0.01 ‘*’ | 0.05 ‘.’ | 0.1 ‘’ | 1 |
| Parameter | Gen | Eta2 |  | 95% | CI |  |
| Protection |  | 0.14 |  | [0.01, | 1.00] |  |
| Year |  | 0.02 |  | [0.00, | 1.00] |  |
| Protection:Year |  | 0.05 |  | [0.00, | 1.00] |  |
| Parameter | partial | Eta2 |  | 95% | CI |  |
| Protection |  | 0.15 |  | [0.02, | 1.00] |  |
| Year |  | 0.03 |  | [0.00, | 1.00] |  |
| Protection:Year |  | 0.06 |  | [0.00, | 1.00] |  |
|  |  |  |  |  |  |  |
| **Year * Island** | | | | | | |
| **aov_island_year <- aov(Dens ~ Island * Year, data = ReefSlope_allyears)** | | | | | | |
|  | Df | Sum Sq | Mean Sq | F Value | Pr(>F) |  |
| Island | 4 | 8100574 | 2025143 | 16.602 | 4.94E-10 | *** |
| Year | 2 | 498574 | 249287 | 2.044 | 0.136 |  |
| Island:Year | 8 | 2317823 | 289728 | 2.375 | 0.0235 | * |
| Residuals | 83 | 10124230 | 121979 |  |  |  |
| Significance codes | 0 ‘***’ | 0.001 ‘**’ | 0.01 ‘*’ | 0.05 ‘.’ | 0.1 ‘’ | 1 |
| Parameter | Gen | Eta2 |  | 95% | CI |  |
| Island |  | 0.38 |  | [0.23, | 1.00] |  |
| Year |  | 0.02 |  | [0.00, | 1.00] |  |
| Island:Year |  | 0.11 |  | [0.00, | 1.00] |  |
| Parameter | partial | Eta2 |  | 95% | CI |  |
| Island |  | 0.44 |  | [0.30, | 1.00] |  |
| Year |  | 0.05 |  | [0.00, | 1.00] |  |
| Island:Year |  | 0.19 |  | [0.02, | 1.00] |  |
|  |  |  |  |  |  |  |
| **Island * Year * Protection** | | | | | | |
| **aov_full <- aov(Dens ~ Island * Year * Protection, data = ReefSlope_allyears)** | | | | | | |
|  | Df | Sum Sq | Mean Sq | F Value | Pr(>F) |  |
| Island | 4 | 8100574 | 2025143 | 15.156 | 7.13E-09 | *** |
| Year | 2 | 498574 | 249287 | 1.866 | 0.1628 |  |
| Protection | 5 | 848370 | 169674 | 1.27 | 0.2873 |  |
| Island:Year | 8 | 2050564 | 256321 | 1.918 | 0.0715 | . |
| Island:Protection | 2 | 212234 | 106117 | 0.794 | 0.4562 |  |
| Year:Protection | 7 | 149694 | 21385 | 0.16 | 0.992 |  |
| Island:Year:Protection | 2 | 228413 | 114206 | 0.855 | 0.43 |  |
| Residuals | 67 | 8952778 | 133624 |  |  |  |
| Significance codes | 0 ‘***’ | 0.001 ‘**’ | 0.01 ‘*’ | 0.05 ‘.’ | 0.1 ‘’ | 1 |
| Parameter | Gen | Eta2 |  | 95% | CI |  |
| Island |  | 0.38 |  | [0.21, | 1.00] |  |
| Year |  | 0.02 |  | [0.00, | 1.00] |  |
| Protection |  | 0.04 |  | [0.00, | 1.00] |  |
| Island:Year |  | 0.1 |  | [0.00, | 1.00] |  |
| Island:Protection |  | 0.01 |  | [0.00, | 1.00] |  |
| Year:Protection |  | 7.11E-03 |  | [0.00, | 1.00] |  |
| Island:Year:Protection |  | 0.01 |  | [0.00, | 1.00] |  |
| Parameter | partial | Eta2 |  | 95% | CI |  |
| Island |  | 0.48 |  | [0.31, | 1.00] |  |
| Year |  | 0.05 |  | [0.00, | 1.00] |  |
| Protection |  | 0.09 |  | [0.00, | 1.00] |  |
| Island:Year |  | 0.19 |  | [0.00, | 1.00] |  |
| Island:Protection |  | 0.02 |  | [0.00, | 1.00] |  |
| Year:Protection |  | 0.02 |  | [0.00, | 1.00] |  |
| Island:Year:Protection |  | 0.02 |  | [0.00, | 1.00] |  |
