# Supplementary figures and images for: Status and trends of giant clam populations demonstrate the effectiveness of village-based protection in American Sāmoa
Source: PeerJ. 2025 Nov 14;13:e20290. doi: 10.7717/peerj.20290 (PMC12622233; doi:10.7717/peerj.20290)

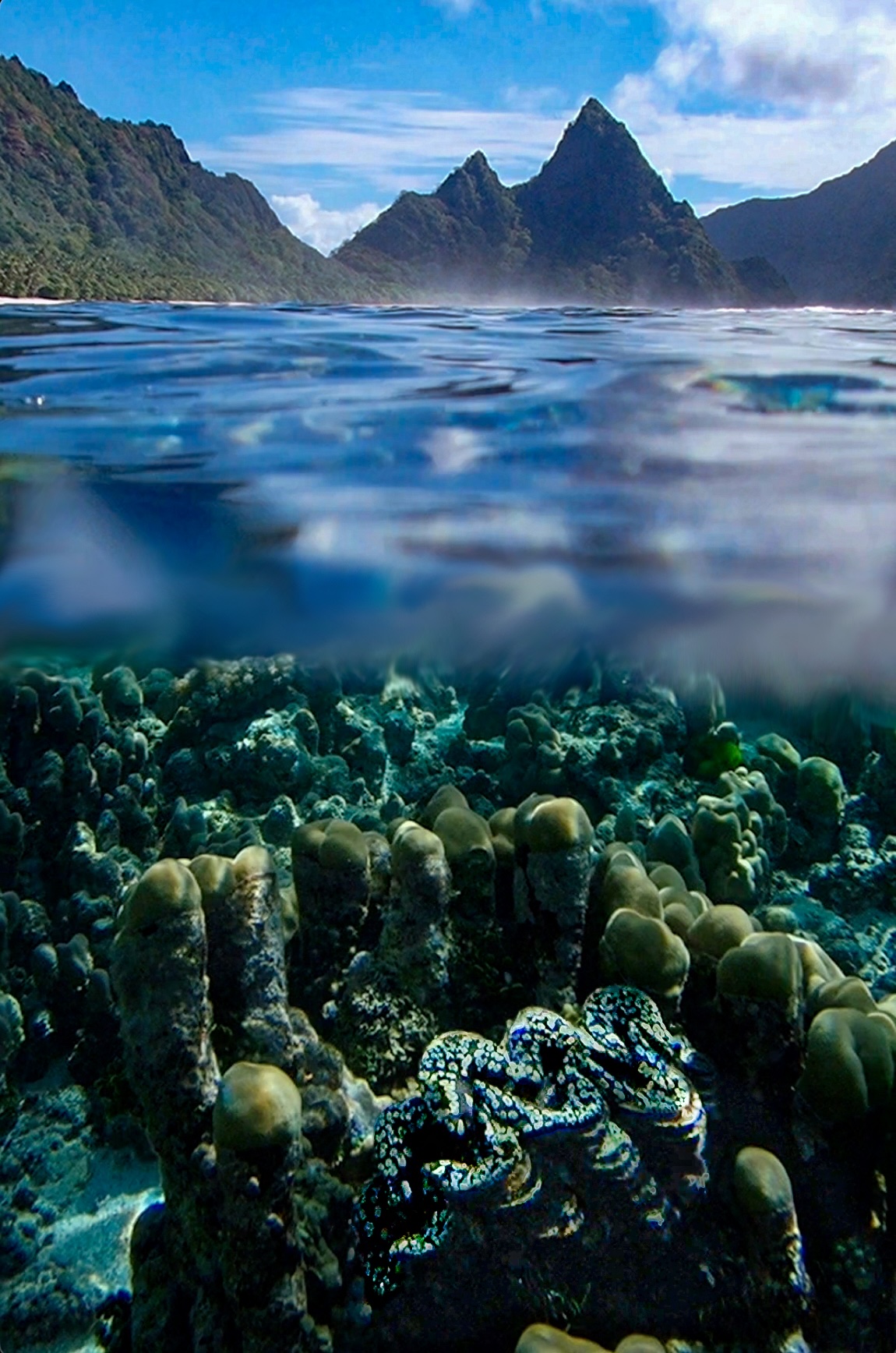

Supplement: Supplemental Information 5 — Photo Credit: Paolo Marra-Biggs [file peerj-13-20290-s005.jpg]

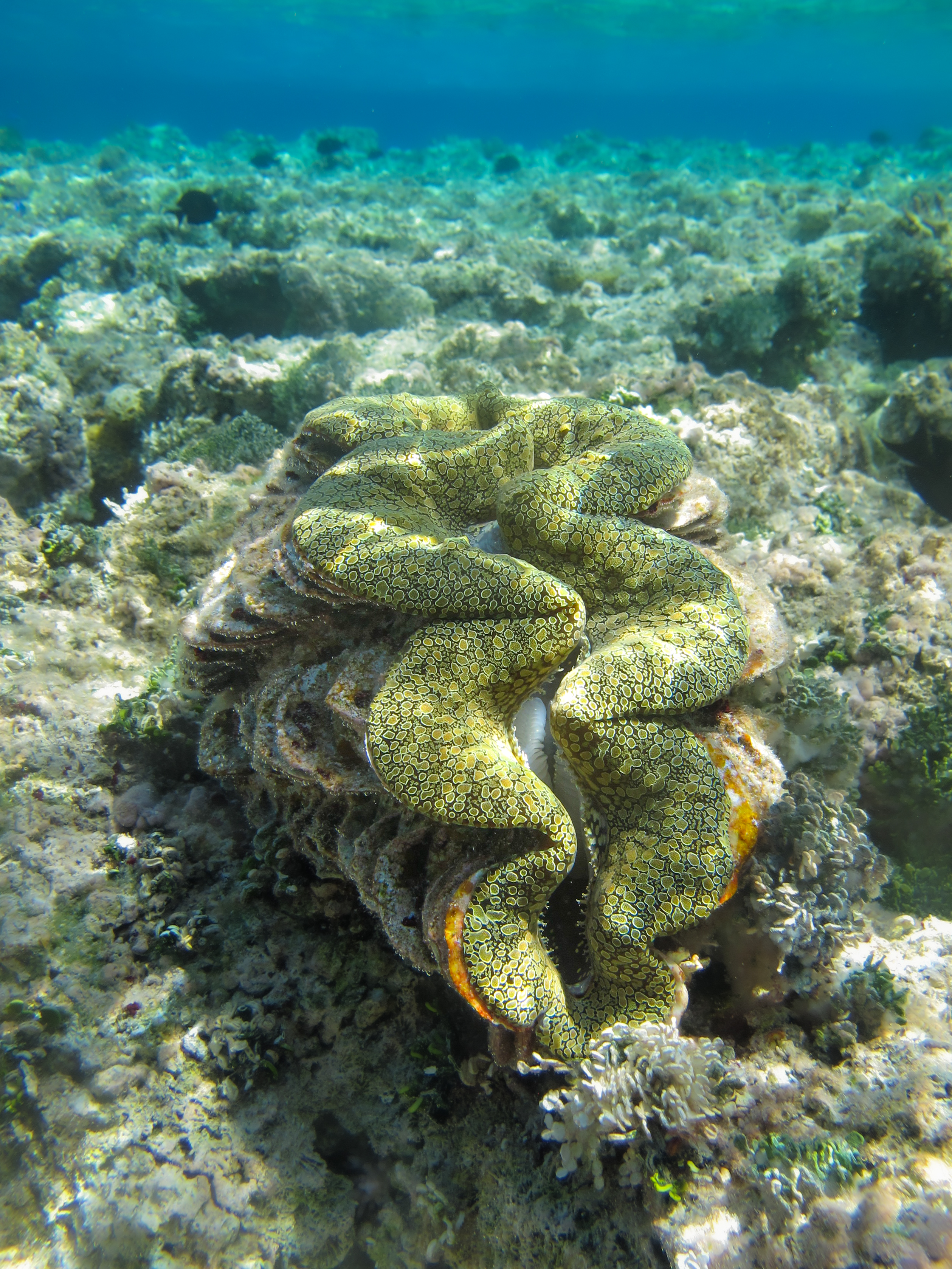

Supplement: Supplemental Information 6 — Photo Credit: Paolo Marra-Biggs [file peerj-13-20290-s006.jpg]

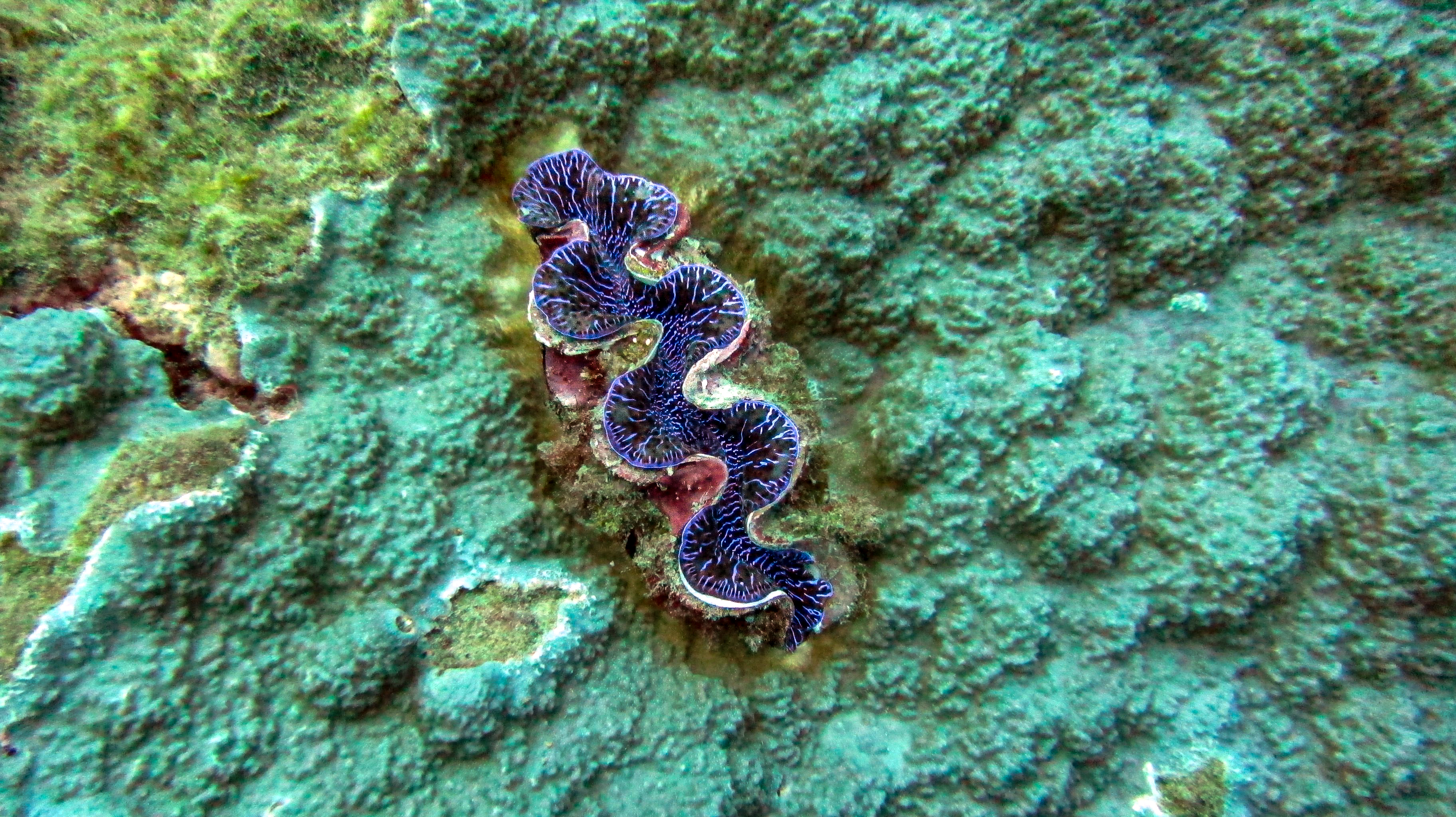

Supplement: Supplemental Information 7 — Photo Credit: Paolo Marra-Biggs [file peerj-13-20290-s007.jpg]

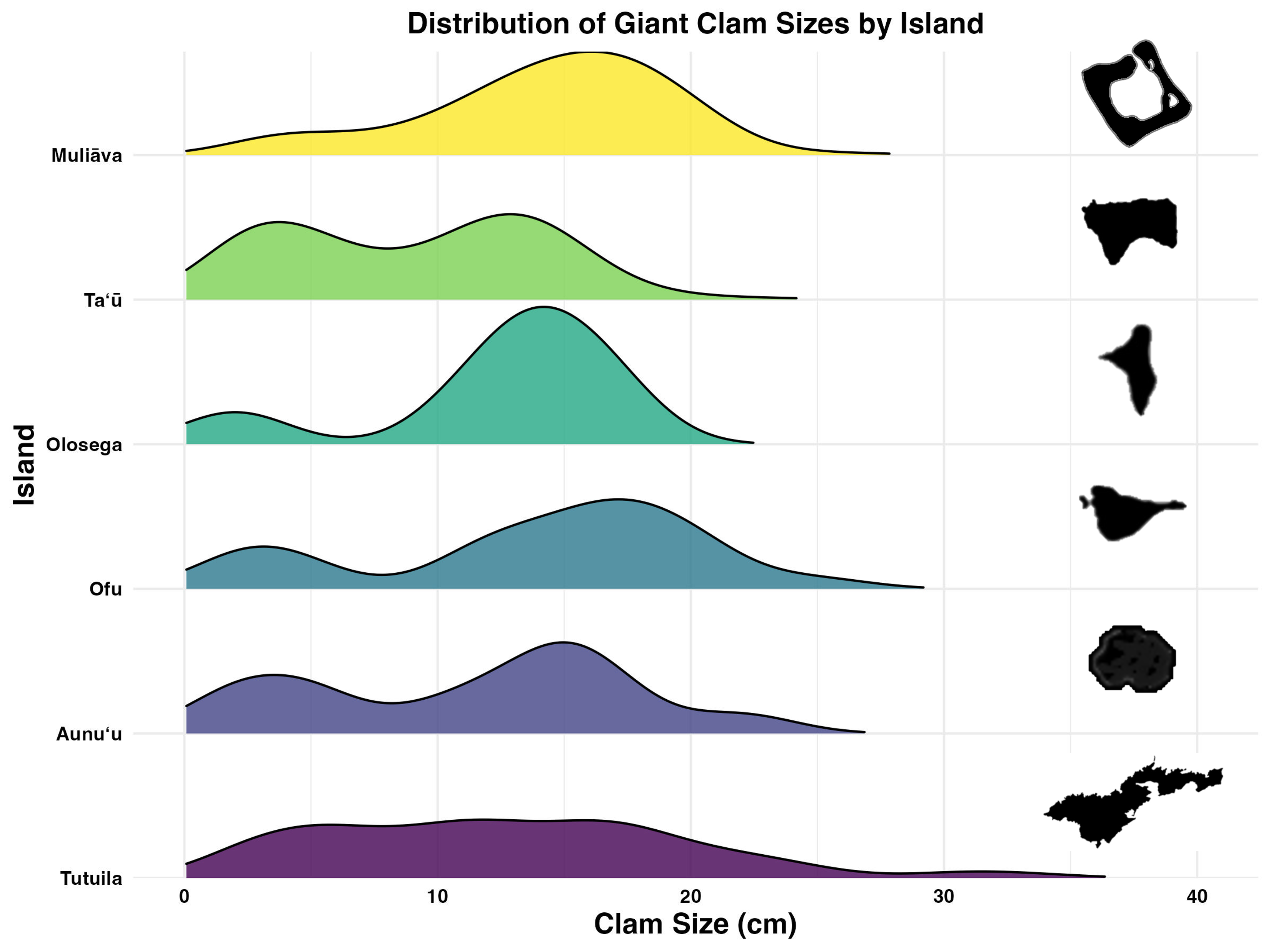

Supplement: Supplemental Information 9 [file peerj-13-20290-s009.png]
